# Reducing Iron Oxide with Ammonia: A Sustainable Path to Green Steel

**DOI:** 10.1002/advs.202300111

**Published:** 2023-03-30

**Authors:** Yan Ma, Jae Wung Bae, Se‐Ho Kim, Matic Jovičević‐Klug, Kejiang Li, Dirk Vogel, Dirk Ponge, Michael Rohwerder, Baptiste Gault, Dierk Raabe

**Affiliations:** ^1^ Max‐Planck‐Institut für Eisenforschung Max‐Planck‐Straße 1 40237 Düsseldorf Germany; ^2^ Department of Metallurgical Engineering Pukyong National University Busan 48513 Republic of Korea; ^3^ School of Metallurgical and Ecological Engineering University of Science and Technology Beijing Beijing 100083 P. R. China; ^4^ Department of Materials Royal School of Mine Imperial College London London SW7 2AZ UK; ^5^ Department of Materials Science and Engineering Korea University Seoul 02841 Republic of Korea

**Keywords:** ammonia, autocatalytic reaction, carbon dioxide emissions, iron oxide, renewable energy, sustainable iron making

## Abstract

Iron making is the biggest single cause of global warming. The reduction of iron ores with carbon generates about 7% of the global carbon dioxide emissions to produce ≈1.85 billion tons of steel per year. This dramatic scenario fuels efforts to re‐invent this sector by using renewable and carbon‐free reductants and electricity. Here, the authors show how to make sustainable steel by reducing solid iron oxides with hydrogen released from ammonia. Ammonia is an annually 180 million ton traded chemical energy carrier, with established transcontinental logistics and low liquefaction costs. It can be synthesized with green hydrogen and release hydrogen again through the reduction reaction. This advantage connects it with green iron making, for replacing fossil reductants. the authors show that ammonia‐based reduction of iron oxide proceeds through an autocatalytic reaction, is kinetically as effective as hydrogen‐based direct reduction, yields the same metallization, and can be industrially realized with existing technologies. The produced iron/iron nitride mixture can be subsequently melted in an electric arc furnace (or co‐charged into a converter) to adjust the chemical composition to the target steel grades. A novel approach is thus presented to deploying intermittent renewable energy, mediated by green ammonia, for a disruptive technology transition toward sustainable iron making.

## Graphical abstract

Here, a sustainable iron‐making process is introduced by directly deploying ammonia as a renewable energy and hydrogen carrier in direct reduction process. The reduction behavior of hematite and the characteristics of its product are investigated. Here a novel approach is provided to deploying intermittent renewable energy mediated by ammonia and using it for a disruptive technology transition toward sustainable iron making.

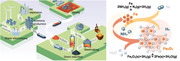

## 1 Introduction

Iron and steel are pillars of global civilization and industrialization, with currently 1.85 billion tons produced per year.^[^ ^1^ ^]^ This staggering amount is forecast to grow up to 2.5–3.0 billion tons by the year 2050.^[^ ^2^ ^]^ As the primary synthesis of iron uses fossil reductants (from coal, coke, and methane) to reduce oxidic ores, the steel industry is currently the largest single producer of carbon dioxide, accounting for ≈7% of the global emissions.^[^ ^3^ ^]^ Disruptive sustainable approaches are urgently needed to address the decarbonization challenge in this sector, enabling a paradigm shift from fossil‐fuel‐based to green‐hydrogen (H_2_)‐based or green‐electricity‐based steel production. This technological transition is the largest untapped leverage against global warming. Several emerging approaches along these lines are currently being matured into industry‐scale sustainable technology solutions to green steel production. Important examples are hydrogen‐based direction reduction (HyDR),^[^ ^4^ ^]^ hydrogen plasma smelting reduction,^[^ ^5^ ^]^ and various electrolysis processes (e.g., molten oxides’ electrolysis,^[^ ^6^ ^]^ molten salt electrolysis,^[^ ^7^ ^]^ waster‐assisted molten salt electrochemical reduction,^[^ ^8^ ^]^ and electrowinning of solid iron from aqueous solutions^[^ ^9^ ^]^). Among these alternatives, the HyDR approach has today reached the highest technology readiness level (TRL 6–8) and is currently being deployed at industrial scale.^[^ ^2^, ^10^ ^]^ In this process, green hydrogen should be ideally used, i.e., hydrogen that has been produced using renewable energy sources, generating water instead of carbon dioxide as redox product.^[^ ^4b,d^ ^]^

Seasonal intermittency of sustainable energy production and the geographic locations of efficient producers mean that the demand and supply of green hydrogen are not in synchrony; both temporally and spatially uncorrelated.^[^ ^11^ ^]^ Thus, renewable energy must be stored and transported, not only regionally but also transcontinentally, like fossil carriers today. However, storage and transport of hydrogen remain a significant challenge due to the high amount of energy required for compressing or liquefying it (e.g., at a high pressure of 350–700 bar or a low temperature of −253 °C, respectively).^[^ ^12^ ^]^ Bringing hydrogen in such transportable conditions costs more than 30% of the embodied chemical energy it delivers.^[^ ^13^ ^]^ Liquid anhydrous ammonia (NH_3_) with a high volumetric hydrogen content (≈121 vs 70.8 kg‐H_2_ m^−3^ in liquid hydrogen at −253 °C) and energy density (4.25 vs 2.81 kWh L^−1^ of liquid hydrogen) is an efficient and cost‐competitive hydrogen and energy storage vectors.^[^ ^14^ ^]^ Ammonia can be liquefied under mild conditions by pressurization (≈8 bar at 25 °C) or refrigeration (−33 °C at 1 bar) for storage and intercontinental transport (via ship, truck, and pipeline), and the logistics is established and cost‐effective.^[^ ^15^ ^]^ Currently, ammonia is synthesized through the Haber–Bosch process by converting hydrogen and nitrogen into ammonia. In this process, hydrogen is mainly produced via steam methane reforming. This fact makes the process of fossil‐fuel‐based ammonia synthesis very carbon dioxide intensive, accounting for ≈1% of the global carbon dioxide emissions.^[^ ^11^, ^16^ ^]^ Yet more sustainable ammonia synthesis pathways are under development to mitigate carbon dioxide emissions in the ammonia industry.^[^ ^11^, ^16^ ^]^ For instance, the electrically driven hybrid Haber–Bosch process (via replacing the steam methane reforming by water electrolysis to obtain green hydrogen and coupling with an ammonia synthesis reactor in the Haber–Bosch process) or direct electrosynthesis using renewable energy (via nitrogen reduction reaction) enables the production of green ammonia.^[^ ^11^, ^16^ ^]^

**Figure 1:**
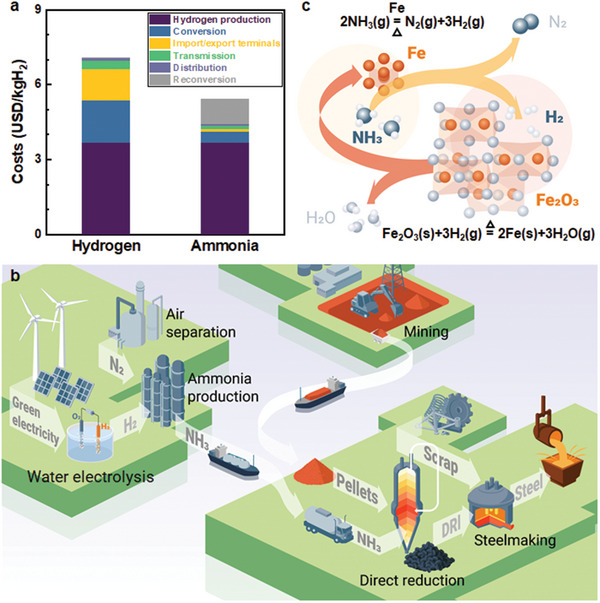
A pathway toward sustainable steel production via ammonia‐based direct reduction. a) Comparison of the predicted costs required for hydrogen and ammonia produced by renewable electricity in 2030, assuming that it is produced in Australia and then transported to Japan (reproduced based on data from the International Energy Agency^[^ ^13a^ ^]^). b) Future steel industry with deploying intermittent renewable energy mediated by green ammonia. c) Autocatalytic reduction of iron oxide by hydrogen released from ammonia cracking during the direct reduction process.

In fact, ammonia has played a key role in global fertilizer production over a century,^[^ ^17^ ^]^ with high technology readiness in production, liquefaction, storage, and transport.^[^ ^14c^ ^]^ Thus, the total costs of delivering ammonia to the end users are predicted to be much lower compared with hydrogen.^[^ ^12^, ^13^, ^18^ ^]^ For example, the total costs of ammonia produced in 2030 using renewable electricity are projected to be ≈5.5 USD kg^−1^ H_2_ (i.e., normalized with respect to the costs of green hydrogen) by the International Energy Agency,^[^ ^13a^ ^]^ assuming in this exemplary calculation that it is produced in Australia and then transported to Japan. In contrast, the predicted costs for green hydrogen yield a much higher value of ≈7.1 USD kg^−1^ H_2_ for the same delivery scenario (**Figure** **1**a; see details in the Supporting Information). It is worth noting that this cost estimation is based on ammonia synthesis via the use of green hydrogen, i.e., the costs for hydrogen production are identical for both assumed scenarios: for the use of hydrogen and for the use of ammonia. The comparison demonstrates that much lower costs apply for the case of ammonia, due to its less costly conversion into the liquid state, storage, and transport. These advantages of ammonia motivate the study of the combination of green‐ammonia‐mediated energy and the hydrogen‐based reduction cycle (with TRL 6–8) for sustainable production of iron and steel.^[^ ^19^ ^]^

Here, we introduce a sustainable iron‐making process by directly deploying ammonia in iron ore reduction (Figure 1b). An important advantage of this approach is that the green ammonia does not need to be cracked into hydrogen and nitrogen using a precious metal catalyst, e.g., ruthenium,^[^ ^20^ ^]^ prior to the reduction process. This absence of a separate catalytic splitting step prior to the reduction reaction makes ammonia economically even more attractive, by avoiding the additional costs of reconversion, with a further cost reduction of ≈18% (i.e., only 4.5 USD kg^−1^ H_2_ for ammonia without reconversion, Figure 1a).^[^ ^13a^ ^]^ We introduce the as‐delivered ammonia into a direct‐reduction reactor (i.e., a static shaft furnace) where solid oxides (e.g., industry‐standard hematite pellets) are exposed to the reducing gas at 700 °C (or higher) to produce direct reduced iron (DRI, also known as sponge iron). This ammonia‐based direct reduction (ADR) process is carbon dioxide free. The sponge iron can be subsequently charged into an electric arc furnace to melt it and to adjust the chemical composition to the target steel grades (Figure 1b). Both direct reduction and electric arc furnaces are industrially available technologies; here, it should be noted that the electric arc can be produced using renewable electricity. Here, we focus on the direct reduction behavior of hematite pellets exposed to ammonia and compare the kinetics with that of HyDR.

## 2 Results and Discussion

### 2.1 Reduction Behavior and Kinetics

**Figure 2:**
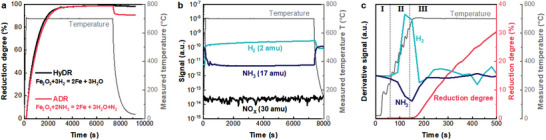
Direct reduction kinetics of hematite pellets with ammonia and the associated evolution of gaseous species. a) Reduction degree for ammonia‐based direct reduction (ADR) and hydrogen‐based direct reduction (HyDR) of hematite pellets at 700 °C. (The reduction degree was obtained from the mass changes.) b) Intensity evolution of hydrogen (H_2_), ammonia (NH_3_), and nitric oxide (NO*_x_*) measured by quadrupole mass spectrometry during ADR. c) Intensity derivatives of hydrogen (H_2_) and ammonia (NH_3_) in the early stage of the reduction.

We performed isothermal reduction on commercial direct‐reduction hematite pellets at 700 °C under a pure ammonia atmosphere using a thermogravimetry setup (“Experimental Section”; Figure S5, Supporting Information). The reduction degree (based on mass loss) for the ADR experiment is shown in **Figure** **2**a and that for HyDR is shown as a reference. The kinetics of the ADR revealed a typical sigmoidal shape known from nucleation‐growth processes, very similar to that for HyDR.^[^ ^4c^ ^]^ The reduction degree of the ADR reached ≈98% during isothermal processing at 700 °C, similar to that of the HyDR process. For the ADR sample, however, a mass gain was observed upon cooling, as indicated by an apparent decline in the reduction degree by ≈7.5% (Figure 2a). This mass gain was attributed to partial in situ nitriding of the reduced iron by ammonia during cooling. The formation of this passivating nitride, i.e., Fe_4_N, on the surface is an important feature of ADR as discussed in the following sections.

The evolution of the mass spectrometry signals of NH_3_, H_2_, and nitrogen oxides (NO*_x_*) during the ADR process is shown in Figure 2b. It is worth noting that no formation of any ozone‐destroying NO*_x_* molecules was observed during ADR. According to the derivatives of the mass spectrometry signals for ammonia and hydrogen, three stages can be distinguished during heating and in the early stage of isothermal reduction (Figure 2c). In stage I (below 350 °C), there was no obvious change in gas composition. In stage II (350–650 °C), a drastic decline in the intensity derivative of ammonia marked the onset of ammonia decomposition above ≈350 °C. Consequently, hydrogen was generated, as shown by a steep increase in the intensity derivative of hydrogen. Stage III began with the onset of the reduction of iron oxides by consuming hydrogen (Figure 2c), indicated by an increase in reduction degree above ≈650 °C (Figure 2c) and the immediate uptick in the corresponding reduction rate (Figure S6, Supporting Information). In fact, iron is a well‐known catalyst for ammonia decomposition.^[^ ^21^ ^]^ Iron oxides (e.g., hematite^[^ ^22^ ^]^ and goethite^[^ ^23^ ^]^) are widely used as precursors, and their surface can be readily reduced into metallic iron. The following reaction pathways have been commonly accepted in the literature: 1) adsorption of an ammonia molecule on a reactive site (NH_3_*, * represents a reactive adsorption site) of the iron surface; 2) stepwise dissociation of nitrogen–hydrogen bonds to form NH*_x_* * (*x* = 1 or 2) and H* species; 3) association of H* and N* to form H_2_* and N_2_*, respectively; and 4) desorption of hydrogen and nitrogen molecules from the iron surface.^[^ ^21b,c^ ^]^ Due to the strong bonding between nitrogen and iron, nitrogen desorption is usually the rate‐limiting step.^[^ ^24^ ^]^ Recently, the underlying atomistic mechanisms and the associated energy barriers of the individual reaction steps have been studied in high detail using density functional theory.^[^ ^21^, ^25^ ^]^ The porous iron formed during direct reduction can thus effectively catalyze ammonia cracking into hydrogen, further promoting the reduction of iron oxides to iron (Figure 1c). Such an autocatalytic reaction offers a path toward further efficiency gains and reductions in both capital expenditure (e.g., equipment costs for ammonia cracking) and operation expenses (e.g., costs of precious metal catalyst). Moreover, nitrogen, a nontoxic, non‐greenhouse gas, as a by‐product of ammonia decomposition can act as a heat carrier in a shaft furnace to maintain the reaction temperature and thus enhance the efficiency for the endothermic reduction of iron oxide with hydrogen.^[^ ^26^ ^]^

### 2.2 Reduction Products

**Figure 3:**
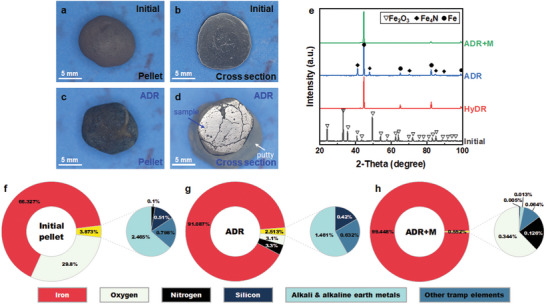
Photographs, phase identification, and chemical analyses of input material and reduction product. a,c) Photographs of pellets before and after reduction at 700 °C for 2 h with ammonia. b,d) The cross‐sectional view (the great circle plane of the spherical samples) of panels (a) and (c), respectively. e) Phase identification from XRD of the initial pellet, HyDR, ADR, and melted‐ADR (ADR+M) samples. f–h) Chemical composition (in wt%) measured by ICP‐OES (see a complete list in Table S1 in the Supporting Information).

The pellets before and after ADR are shown in **Figure** **3**a–d. After ADR, the surface of the pellet revealed a bluish color (Figure 3c) in contrast to the initial pellet showing a reddish surface (Figure 3a). The metallic luster became visible in the cross section of the spherical ADR sample (Figure 3d), indicating the reduction of hematite to metallic iron. The X‐ray diffraction (XRD) measurement confirmed that the ADR product comprised ≈60 wt% body‐cantered‐cubic iron and ≈40 wt% Fe_4_N nitride (Figure 3e). In contrast, the HyDR reference product was essentially pure iron. The bulk chemical analysis obtained using inductively coupled plasma optical emission spectrometry (ICP‐OES) further showed a high iron content of up to ≈91 wt% in the ADR sample (Figure 3g). A minor amount of remaining oxygen of ≈3.1 wt% was detected and mostly likely bound within inert gangue oxides (e.g., silicon, magnesium, and aluminum oxides), which were not readily reduced under such reduction conditions.^[^ ^4c^ ^]^ The measurements also showed a nitrogen content of ≈3.3 wt% in the ADR product (Figure 3g). Such a value concurred well with the apparent change in the reduction degree by ≈7.5% measured by thermogravimetry during cooling (Figure 2a), as the latter was supposed to constitute ≈3.13 wt% nitrogen in the reduced iron (see calculation in the Supporting Information). These numbers testified that the nitrogen in the ADR product mostly stemmed from nitriding during cooling rather than during the reduction process at 700 °C.

**Figure 4:**
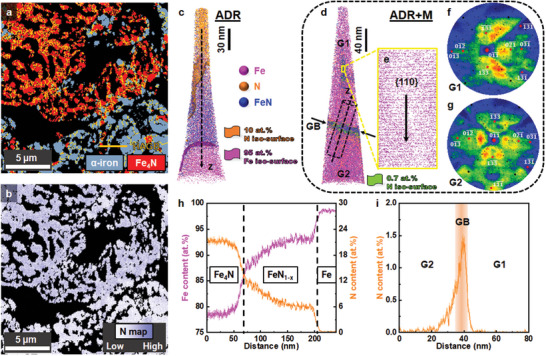
Micro‐ and nanostructures of the ADR and the melted‐ADR (ADR+M) samples. a) Phase map of ADR sample constructed by electron backscatter diffraction (HAGB: high‐angle grain boundaries with misorientation angle larger than 10° and the black regions representing pores). b) The corresponding nitrogen map of panel (a) measured by energy‐dispersive X‐ray spectroscopy. c,d) 3D reconstructions of APT specimens taken from the ADR and ADR+M samples, respectively, revealing local enrichment of nitrogen. e) Thin slice through the APT dataset (*d*), showing {110} atomic planes of body‐centered‐cubic iron. f,g) Point density maps of the upper and lower parts of the APT specimen (*d*), confirming the different crystallographic orientations of grain 1 (G1) and grain 2 (G2). h,i) 1D compositional profiles along the longitudinal direction of the dash arrows in panels (c) and (d), respectively.

The micro‐ and nanostructures of the ADR product are displayed in **Figure** **4**. It assumed a porous form (Figure 4a, the black regions represent pores), as commonly observed in HyDR‐produced iron.^[^ ^4^, ^27^ ^]^ Such a porous structure is mainly due to the net volume loss of the material when the oxygen gets removed during the reduction process, through a sequence of vacancy formation, vacancy condensation into nanopores, and capillary‐driven pore coarsening.^[^ ^4^, ^28^ ^]^ The phase map constructed by electron backscatter diffraction (EBSD) further confirmed the *α*‐iron and Fe_4_N dual‐phase microstructure in the ADR reduction product. The corresponding elemental map of nitrogen probed by energy‐dispersive X‐ray spectroscopy (Figure 4b) agreed well with the spatial distribution of Fe_4_N (Figure 4a). Near‐atomic scale compositional mapping of the ARD product was obtained from atom probe tomography (APT) (Figure 4c–i). Compositional analysis from the 3D elemental distribution across the interface between reduced iron and Fe_4_N nitride indicated that the Fe_4_N nitride contained ≈21 at% nitrogen (Figure 4h), close to its expected stoichiometry. A transition region containing ≈6 at% nitrogen (Figure 4h) extended over 100 nm into the pure iron below the Fe_4_N (Figure 4c).

The nitride formation is another key advantage of ADR, as nitriding improves the aqueous corrosion resistance of iron.^[^ ^29^ ^]^ The nitride passivated the otherwise highly active reduced iron, offering a safety‐critical benefit for handling and logistics. Otherwise, for the downstream processing of the reduced material, the porous sponge iron is prone to re‐oxidation and strong exothermic reactions with oxygen or moisture due to its high surface‐to‐volume ratio (typically above 40 vol% porosity^[^ ^4c^ ^]^). Thus, the sponge iron produced by HyDR must be compacted into hot briquetted iron to reduce the porosity for shipping and handling, which is not necessary with ADR.

The protective nitride phase was completely dissolved and removed after melting, as measured by XRD (Figure 3e). Thus, such a melting process resulted in a final material with a very high concentration of iron ≈99.4% and only 0.1–0.15 wt% nitrogen retained in the iron (Figure 3h). Figure 4d is the APT analysis across a grain boundary in the melted sample after solidification. The different crystallographic orientations of the grains were confirmed by the point density maps^[^ ^30^ ^]^ (Figure 4f,g). The {110} atomic planes of *α*‐iron (Figure 4e) were readily imaged as well. The 1D concentration profile (Figure 4i) across the boundary evidenced that nitrogen was primarily confined to grain boundaries, with a peak composition of 1.5 at%. Segregation of nitrogen to microstructural defects is expected from its low solubility in iron at low temperatures (e.g., ≈0.05–0.06 wt% at 500 °C) during cooling.^[^ ^31^ ^]^

## 3 Conclusion

In summary, ADR is kinetically as effective for producing green iron as HyDR at 700 °C. The direct utilization of ammonia in the reduction process offers a process shortcut, alleviating the need for a preliminary ammonia cracking step into hydrogen and nitrogen. During the redox reaction, the gradually generated porous iron further catalyzes the decomposition of ammonia at elevated temperatures, to release hydrogen for the reduction of iron oxides. This autocatalytic reaction provides a path to further efficiency gains and cost reductions. The in situ nitriding from the process offers protection of the pure iron against environmental degradation that otherwise requires dedicated additional process steps that are energetically and logistically costly. Such a protective nitride phase can be completely dissolved and removed during a subsequent melting process. Thus, ADR provides a novel approach to deploying intermittent renewable energy for an unprecedented and disruptive technology transition toward sustainable metallurgical processes. With these benefits, it connects two of the currently most greenhouse gas intense industries (namely, steel and ammonia production industries) and opens a pathway to render them more environmentally benign and sustainable. At the same time, it can eliminate logistic and energetic disadvantages associated with the use of pure hydrogen, when it needs to be transported.

## 4 Experimental Section

### Materials

Commercial direct‐reduction hematite pellets provided by Huasco Pellet Plant were used in the present study. The pellets had a diameter of ≈11 mm and a weight of ≈2.7 g. The chemical composition of the pellets is listed in Table S1 (Supporting Information).

### Direct Reduction and Sample Preparation

The pellets were exposed to ammonia (purity = 99.999%) and hydrogen (purity = 99.999%) gases in a thermogravimetric (TG) configuration (Figure S5, Supporting Information).^[^ ^32^ ^]^ The samples were heated up with infrared light to 700 °C with a ramping rate of 5 °C s^−1^ and then held isothermally for 2 h. After the isothermal treatment, the power of the TG furnace was switched off and samples were cooled in the furnace. The temperature profile was measured by a thermocouple inserted into the center of a reference pellet, and the result is shown in Figure 2a. The flow rate of gases was set as 10 L h^−1^ during the entire experiment. The mass loss of a pellet was continuously monitored by the thermal balance during the reduction experiment. The reduction degree was determined from the experimental mass loss divided by the theoretical mass loss, considering Fe_2_O_3_ being fully reduced into Fe. A quadrupole mass spectrometer with a quartz capillary gas inlet was attached to the TG setup for analyzing gaseous compounds (e.g., NH_3_, N_2_, H_2_, H_2_O, and NO*_x_*). A disk sample with a thickness of ≈1 mm was prepared from the center of the spherical pellet for microstructure analysis. To investigate the phase stability and chemical composition of the reduced pellet after melting, the ADR pellets were melted in an arc melting furnace (Edmund Bühler GmbH) in the Ar atmosphere under 900 mbar for 65 s.

### Chemical Analysis

The metallic elements of the samples were measured by ICP‐OES. The oxygen content was measured in a reduction fusion (in a helium atmosphere), and carbon and sulfur contents via combustion by infrared absorption spectroscopy. The contents of nitrogen and hydrogen were measured by thermal conductivity measurement in a reduction fusion (in a helium and nitrogen atmosphere, respectively).

### X‐Ray Diffraction

The phase constituents of the samples were identified by XRD using a RIKAKU SMARTLAB 9KW diffractometer with Cu K*α* radiation (*λ* = 1.54059 Å). The scanning range 2*θ* was from 10° to 100° with a scanning step of 0.01° and a scanning speed of 2° min^−1^.

### Electron Backscatter Diffraction and Energy‐Dispersive X‐Ray Spectroscopy

EBSD measurement with chemical indexing (Chi scan assisted by energy‐dispersive X‐ray spectroscopy) was performed using a Zeiss Sigma scanning electron microscope with an accelerating voltage of 30 kV.

### Atom Probe Tomography

The APT specimens were prepared in an FEI Helios NanoLab600i dual‐beam focused ion beam/scanning electron microscopy instrument by lift‐out and annular milling procedures. For the APT measurements, the Cameca LEAP 5000XR instrument was used to collect the data in laser‐pulsing mode at a wavelength of 355 nm. The laser energy and pulse frequency were 40 pJ and 200 kHz, respectively. During the APT measurements, the temperature in the analysis chamber was maintained at 50 K. The reconstruction of the 3D atom maps and data analyses were carried out using the commercial software AP Suite 6.1.

## Conflict of Interest

The authors declare no conflict of interest.

## Supporting information

Supporting Information

## Data Availability

The data that support the findings of this study are available from the corresponding author upon reasonable request.
