# Supplementary material for: Reducing Iron Oxide with Ammonia: A Sustainable Path to Green Steel
Source: Adv Sci (Weinh). 2023 Mar 30;10(16):2300111. doi: 10.1002/advs.202300111 (PMC10238216; doi:10.1002/advs.202300111)
Supplement: Supplementary file 1 — Supporting Information [file ADVS-10-2300111-s001.pdf]

ADVANCED SCIENCE Open Access

Supporting Information

for *Adv. Sci.*, DOI 10.1002/advs.202300111

Reducing Iron Oxide with Ammonia: A Sustainable Path to Green Steel

Yan Ma, Jae Wung Bae, Se-Ho Kim, Matic Jovičević-Klug, Kejiang Li, Dirk Vogel, Dirk Ponge, Michael Rohwerder, Baptiste Gault and Dierk Raabe**

Supplementary information

Reducing iron oxide with ammonia: A sustainable path to green steel

Yan Ma[1,5,✉], Jae Wung Bae[1,2,5], Se-Ho Kim[1], Matic Jovičević-Klug[1], Kejiang Li[3], Dirk Vogel[1], Dirk Ponge[1], Michael Rohwerder[1], Baptiste Gault[1,4], Dierk Raabe[1,✉]

[1] Max-Planck-Institut für Eisenforschung, Max-Planck-Straße 1, 40237 Düsseldorf, Germany

[2] Department of Metallurgical Engineering, Pukyong National University, Busan 48513, Republic of Korea

[3] School of Metallurgical and Ecological Engineering, University of Science and Technology Beijing, Beijing 100083, PR China

[4] Department of Materials, Royal School of Mine, Imperial College London, London SW7 2AZ, UK

[5] These authors contributed equally: Yan Ma, Jae Wung Bae

✉ Corresponding authors: d.raabe@mpie.de (D.R.); y.ma@mpie.de (Y.M.)

Supplementary Information

Table of Contents

1. Thermodynamic assessment of the reduction of hematite with hydrogen and ammonia .. 3

2. Economic advantages of using ammonia as a hydrogen carrier for ironmaking 5

3. Chemical analysis of the initial and reduced samples 9

4. Calculation of expected mass gain due to nitriding upon cooling 10

5. Thermogravimetry setup for gaseous reduction 11

6. Reduction rate of direct reduction of hematite with hydrogen and ammonia 12

7. References 13

1. Thermodynamic assessment of the reduction of hematite with hydrogen and ammonia

The thermodynamics of the overall reaction of hematite with hydrogen and ammonia was calculated using the FactSage 8.2 software package with FactPS (2022) database. Ammonia-based direct reduction (ADR) reveals much more negative Gibbs free energy compared with hydrogen-based direct reduction (HyDR), as shown in **Supplementary Fig. 1**a. ADR could occur at a much lower temperature (~300 °C), while HyDR is supposed to take place above ~550 °C. These facts suggest that ADR is thermodynamically more favourable than HyDR.

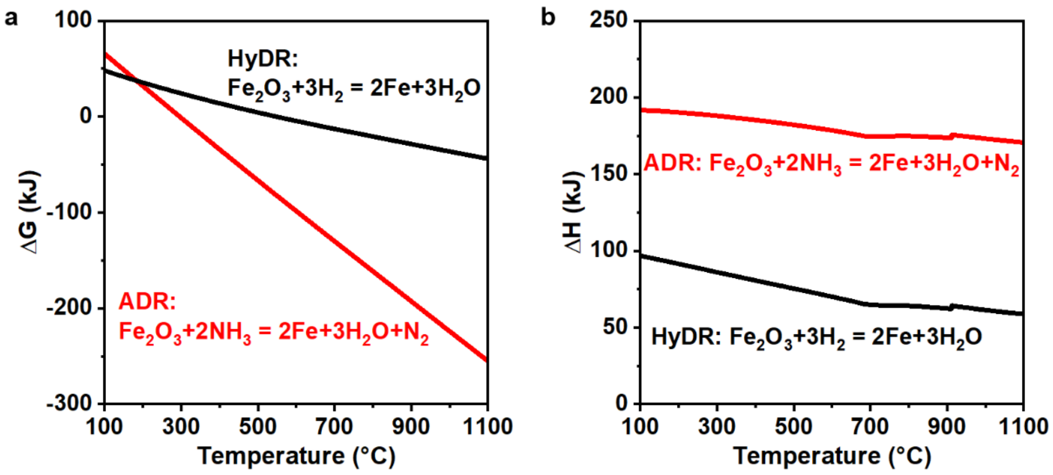

Supplementary Fig. 1 | Thermodynamic assessment of the direct reduction of hematite with hydrogen (HyDR) and ammonia (ADR). a, Reaction Gibbs free energy (ΔG) as a function of temperature. **b**, Reaction enthalpy (ΔH) as a function of temperature.

Due to thermodynamic advantages, ADR can occur at a lower partial pressure of ammonia compared with that of hydrogen in HyDR (**Supplementary Fig. 2**). To reduce hematite to iron, the required partial pressure of ammonia is as low as zero in a temperature range of 500-1100 °C, which is the conventional operating temperature of gaseous reduction processes. In contrast, the partial pressure of hydrogen for HyDR in this temperature range has to be above 0.2~0.35. This thermodynamic benefit of the ADR process is essentially important for its

industrial application. Keeping a high partial pressure of reducing gas is normally very difficult in the reduction process, as the reducing gas is consumed very fast. Alternatively, overblowing reducing gas is necessary to maintain the required high partial pressure[1], which leads to low gas utilization efficiency and requires additional collection and recycling processes of off-gas.

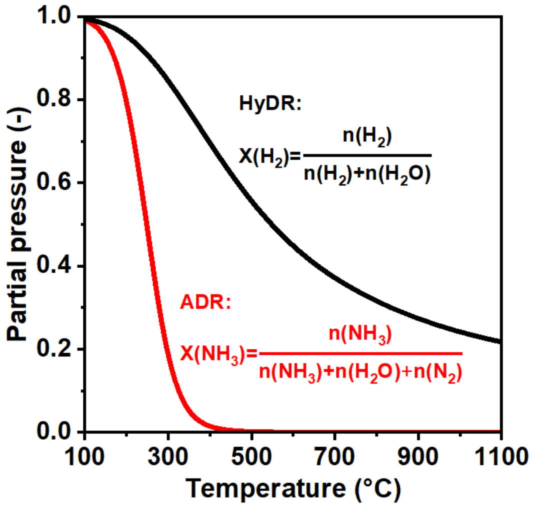

Supplementary Fig. 2 | Equilibrium partial pressure of reductants X(H_2) and X(NH_3) as a function of temperature.

Compared with HyDR, additional heat is required for ADR to crack the ammonia molecules into hydrogen and nitrogen (**Supplementary Fig. 1**b). Nevertheless, some heat is stored in the nitrogen molecules. As nitrogen can act as an energy carrier to overcome the temperature drop when hydrogen reacts with iron oxides (endothermic), heat storage in nitrogen can be beneficial for the overall reduction process[2].

2. Economic advantages of using ammonia as a hydrogen carrier for ironmaking

Because of the high volumetric hydrogen content (~121 kg-H_2/m^3) and energy density (4.25 kWh/L) of ammonia, it is considered one of the most promising hydrogen carriers[3-7]. Currently, the global production of ammonia accounts for more than 180 million tons per year[8]. About 85% of ammonia is mainly used for fertilizer production in the agriculture industry[9-11]. The high technology readiness in the production, liquefaction, storage, and transport of ammonia supports its large-volume trade globally. The Haber-Bosch process is the main method to produce ammonia, which is based on a catalytic reaction to combine hydrogen and nitrogen gases. Currently, hydrogen gas is mainly produced using fossil-fuel feedstocks (mainly natural gas and coal), and nitrogen is delivered through an air separation unit[8]. The fossil-fuel-based ammonia production process is emission-intensive, which leads to 450 million tons of CO_2 emissions annually and accounts for more than 1% of global anthropogenic emissions[12]. To mitigate CO_2 emissions, green ammonia synthesis has been pursued based on green hydrogen production through water electrolysis[11-13].

The value chain of hydrogen and ammonia production, storage, and transport for the future steel industry is shown in **Supplementary Fig. 3**. Ammonia reveals significant energetical and economic advantages over hydrogen in terms of conversion, storage, and transport[14,15]. In the following parts, the cost for the storage and transport of hydrogen directly and via ammonia will be compared and discussed. Considering ammonia synthesis based on green hydrogen production, the cost for hydrogen production is identical for both hydrogen and ammonia routes.

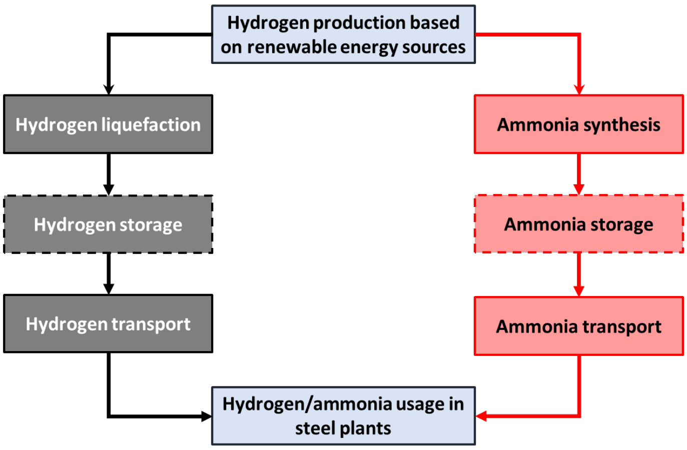

Supplementary Fig. 3 | Hydrogen and ammonia production, storage, and transport value chain for the steel industry.

After production, hydrogen can be either converted to a liquid hydrogen or liquid ammonia for storage and transport. Compared with liquid ammonia storage (at -33°C), liquid hydrogen storage requires a very low temperature down to -253 °C. The latter is a very energy-intensive process. The energy required for liquifying hydrogen and converting hydrogen to liquid ammonia is ~43 MJ/kgH_2 and 9 MJ/kgH_2, respectively[15]. Also, a recent study demonstrated a novel non-equilibrium thermochemical synthesis approach of ammonia towards improved syntheses rate, catalyst stability, and energy efficiency, which may further reduce the cost of energy for ammonia conversion[16]. After conversion, the local storage of liquid hydrogen consumes additional energy to maintain a very low temperature in the storage vessels. Bartels[15] estimated a cost (both conversion and storage, including the capital cost) for hydrogen storage to be ~2 USD/kgH_2, while only 0.86 USD/kgH_2 for ammonia storage, for 15 days of storage. It is worth noting that the cost of storage is proportional to storage time.

Both hydrogen and ammonia can be transported via pipeline, ship, truck, and rail. In general, hydrogen transport is much more expensive than ammonia, as the former has a low volumetric energy density and requires a very low temperature or a high pressure during transportation[14].

Pipelines and ships are considered to be the major transport methods in the future, potentially allowing for large-scale overseas transport capacity[14]. In both cases, the estimated cost for transporting ammonia is much lower than hydrogen, as shown in **Supplementary Fig. 4**.

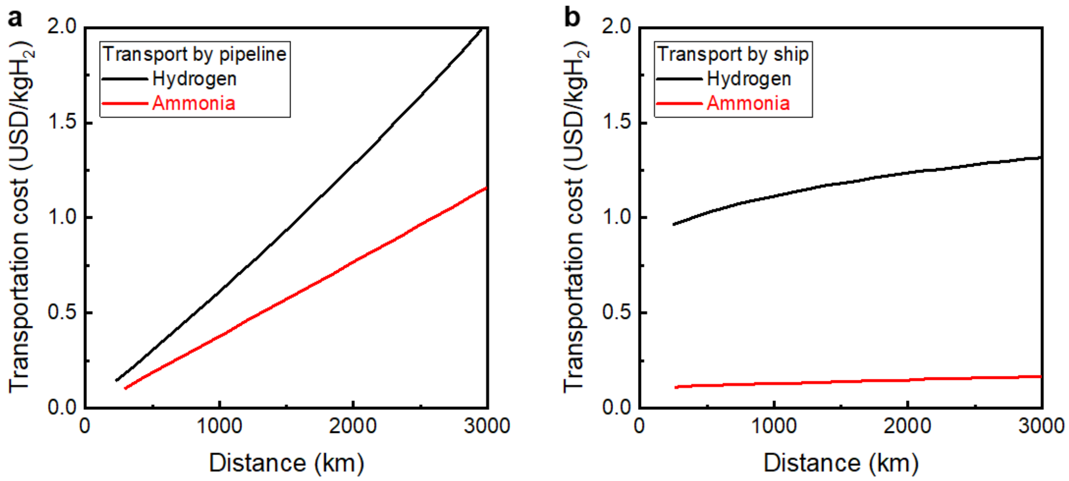

Supplementary Fig. 4 | Comparison of transportation cost of hydrogen and ammonia: a, Transport by pipeline. **b**, Transport by ship[14].

For ammonia, the technology and infrastructure for transportation are already mature, because of its wide utilization in the agriculture industry. For instance, there are more than 10000 storage sites available for ammonia in the USA[17]. Several commercial pipeline systems exist in the world to transport ammonia. For example, there is a 4830 km ammonia pipeline system in operation in the USA[15] and a 2471 km ammonia pipeline system running from Russia to Ukraine[8]. In comparison, the hydrogen pipeline system is very limited and there is only a 719 km hydrogen pipeline system in the USA[15]. Therefore, lots of new infrastructures (*e.g.*, pipelines, transport tanks, as well as import and export terminals) are required to be developed if hydrogen will be transported directly. Such construction will consume a lot of steel, which could lead to a substantial rebound effect.

Last but not least, in the steel industry, ammonia can be used directly in the ironmaking process (as shown in this study), without a separate and costly reconversion procedure to crack

ammonia into hydrogen. This advantage makes ammonia even more attractive by avoiding an additional cost of ~1 USD/kgH_2 for the reconversion process.

3. Chemical analysis of the initial and reduced samples

Supplementary Table 1 summarizes the chemical composition of the initial direct-reduction (DR) pellet, the sample produced by ammonia-based direct reduction (ADR), and melted iron (ADR+M).

Supplementary Table 1 | Chemical composition of the initial direct-reduction (DR) pellets provided by Huasco Pellet Plant, sponge iron produced by direct reduction with ammonia (ADR), and melted iron (ADR+M) (in wt.%).

Sample	Al	C	Ca	H	K	Mg	Mn	N
DR pellet	0.88-0.93	0.027-0.076	0.70-0.83	<0.001	0.017	0.72-0.77	0.38-0.41	<0.1
ADR	0.305	0.023-0.046	0.70	0.013-0.016	0.015	0.426	0.111	1.8-4.8
ADR+M	<0.001	0.0051	0.0043	<0.001	<0.002	0.0037	0.0019	0.099-0.152

Sample	Na	O	P	S	Si	Ti	V	Fe
DR pellet	0.018	29.8	0.016	<0.001	0.51	0.086-0.121	0.215-0.232	Bal.
ADR	0.015	2.92-3.28	0.009	0.0020	0.38-0.46	0.111	0.349	Bal.
ADR+M	<0.002	0.344	<0.005	0.0024	<0.005	0.019	0.030	Bal.

4. Calculation of expected mass gain due to nitriding upon cooling

Mass of an initial pellet (W_{DR}) for ammonia-based direct reduction:

$$W_{DR} = 2747.8\, mg$$

Mass of iron in the pellet (W_{Fe}):

$$W_{Fe} = W_{DR} \times F_{Fe} = 2747.8\, mg \times 66.327\% = 1822.5\, mg$$

where, F_{Fe} is the iron content in the pellet measured by ICP-OES (Supplementary Table 1).

When the iron is all bound with oxygen as hematite (Fe_2O_3), the mass of hematite ($W_{Fe_2O_3}$, upper boundary):

$$W_{Fe_2O_3} = \frac{W_{Fe}}{f_{Fe}} = \frac{1822.5mg}{0.7} = 2603.6\, mg$$

where, f_{Fe} is the theoretical iron fraction in pure hematite.

The theoretical mass loss due to the removal of oxygen in hematite ($W_O^{Fe_2O_3}$, upper boundary):

$$W_O^{Fe_2O_3} = W_{Fe_2O_3} \times f_O = 2603.6\, mg \times 0.3 = 781.1\, mg$$

where, f_O is the theoretical oxygen fraction in pure hematite.

The change in reduction degree measured during cooling is 7.45%, thus, the mass gain ΔW_N is supposed to be (upper boundary by assuming all nitrogen is bound with iron as nitrides):

$$\Delta W_N = W_O^{Fe_2O_3} \times 7.45\% = 58.2\, mg$$

Therefore, the nitrogen content in the final reduced iron can be calculated:

$$F_N = \frac{\Delta W_N}{W_{Fe} \times 98.7\% + \Delta W_N} = \frac{58.2\, mg}{1822.5\, mg \times 98.7\% + 58.2\, mg} \times 100\% = 3.13\, wt.\%$$

This number is in excellent agreement with the nitrogen content measured by ICP-OES in the ADR sample, the mean value of which is 3.3 wt.%. (98.7% is the reduction degree.)

5. Thermogravimetry setup for gaseous reduction

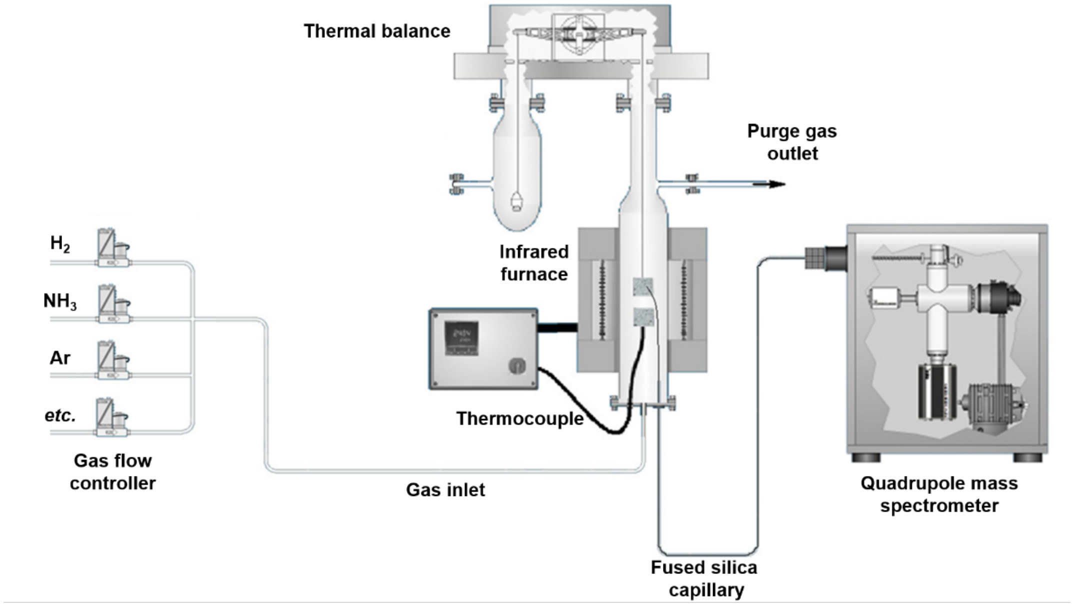

Supplementary Fig. 5 | Thermogravimetry setup coupled with a quadrupole mass spectrometer for direct reduction experiments.

6. Reduction rate of direct reduction of hematite with hydrogen and ammonia

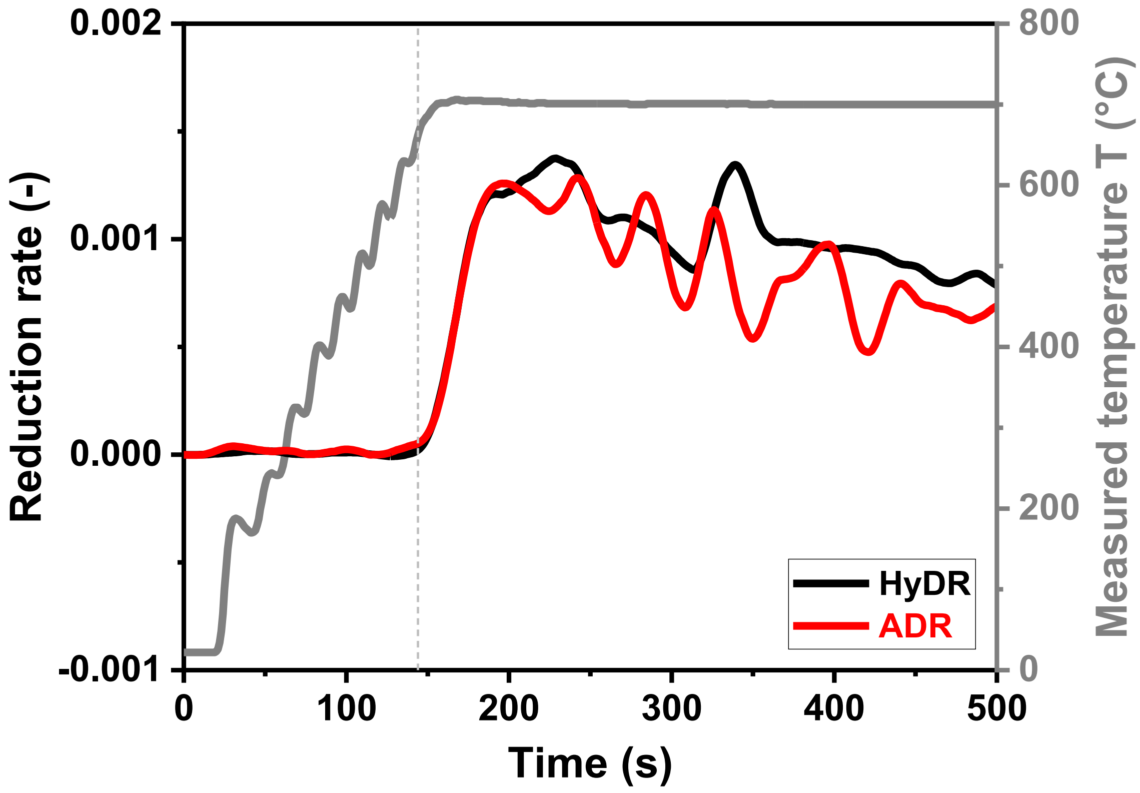

Supplementary Fig. 6 | The reduction rate (a derivative of reduction degree) as a function of time in the early stage of reduction at 700 °C. The dashed line marks the onset of reduction of the hematite pellet. A positive value of the reduction rate indicates an increase in reduction degree.

7. References

1 Spreitzer, D. & Schenk, J. Reduction of Iron Oxides with Hydrogen—A Review. *Steel Research International* **90**, 1900108 (2019). https://doi.org:10.1002/srin.201900108

2 Wang, R. R., Zhao, Y. Q., Babich, A., Senk, D. & Fan, X. Y. Hydrogen direct reduction (H-DR) in steel industry—An overview of challenges and opportunities. *Journal of Cleaner Production* **329**, 129797 (2021). https://doi.org:10.1016/j.jclepro.2021.129797

3 He, T., Pachfule, P., Wu, H., Xu, Q. & Chen, P. Hydrogen carriers. *Nature Reviews Materials* **1**, 16059 (2016). https://doi.org:10.1038/natrevmats.2016.59

4 Xie, P. *et al.* Highly efficient decomposition of ammonia using high-entropy alloy catalysts. *Nature Communications* **10**, 4011 (2019). https://doi.org:10.1038/s41467-019-11848-9

5 Guo, J. & Chen, P. Catalyst: NH3 as an Energy Carrier. *Chem* **3**, 709-712 (2017). https://doi.org:https://doi.org/10.1016/j.chempr.2017.10.004

6 Mazloomi, K. & Gomes, C. Hydrogen as an energy carrier: Prospects and challenges. *Renewable and Sustainable Energy Reviews* **16**, 3024-3033 (2012). https://doi.org:https://doi.org/10.1016/j.rser.2012.02.028

7 Christensen, C. H., Johannessen, T., Sørensen, R. Z. & Nørskov, J. K. Towards an ammonia-mediated hydrogen economy? *Catalysis Today* **111**, 140-144 (2006). https://doi.org:https://doi.org/10.1016/j.cattod.2005.10.011

8 IEA. Ammonia Technology Roadmap. (International Energy Agency, Paris, 2021. <https://www.iea.org/reports/ammonia-technology-roadmap>.

9 Smil, V. Detonator of the population explosion. *Nature* **400**, 415-415 (1999). https://doi.org:10.1038/22672

10 Erisman, J. W., Sutton, M. A., Galloway, J., Klimont, Z. & Winiwarter, W. How a century of ammonia synthesis changed the world. *Nature Geoscience* **1**, 636-639 (2008). https://doi.org:10.1038/ngeo325

11 Lee, B. *et al.* Pathways to a Green Ammonia Future. *ACS Energy Letters* **7**, 3032-3038 (2022). https://doi.org:10.1021/acsenergylett.2c01615

12 Smith, C., Hill, A. K. & Torrente-Murciano, L. Current and future role of Haber–Bosch ammonia in a carbon-free energy landscape. *Energy & Environmental Science* **13**, 331-344 (2020). https://doi.org:10.1039/c9ee02873k

13 Giddey, S., Badwal, S. P. S., Munnings, C. & Dolan, M. Ammonia as a Renewable Energy Transportation Media. *ACS Sustainable Chemistry & Engineering* **5**, 10231-10239 (2017). https://doi.org:10.1021/acssuschemeng.7b02219

14 IEA. The Future of Hydrogen. (International Energy Agency, 2019. <https://www.iea.org/reports/the-future-of-hydrogen>.

15 Bartels, J. R. *A feasibility study of implementing an Ammonia Economy* Master of Science thesis, Iowa State University, (2008).

16 Dong, Q. *et al.* Programmable heating and quenching for efficient thermochemical synthesis. *Nature* **605**, 470-476 (2022). https://doi.org:10.1038/s41586-022-04568-6

17 Ammonia: zero-carbon fertiliser, fuel and energy store. Report No. 978-1-78252-448-9, (The Royal Society, 2020. <https://royalsociety.org/green-ammonia>.
